# Burst-Enhanced Super-Resolution Network (BESR)

**DOI:** 10.3390/s24072052

**Published:** 2024-03-23

**Authors:** Jiaao Li, Qunbo Lv, Wenjian Zhang, Yu Zhang, Zheng Tan

**Affiliations:** 1 Aerospace Information Research Institute, Chinese Academy of Sciences, No. 9 Dengzhuang South Road, Haidian District, Beijing 100094, China; lijiaao21@mails.ucas.ac.cn (J.L.); lvqunbo@aoe.ac.cn (Q.L.); zhangwenjian21@mails.ucas.ac.cn (W.Z.); zhangyu20e@mails.ucas.ac.cn (Y.Z.); 2 School of Optoelectronics, University of Chinese Academy of Sciences, No. 19(A) Yuquan Road, Shijingshan District, Beijing 100049, China; 3 Department of Key Laboratory of Computational Optical Imagine Technology, Chinese Academy of Sciences, No. 9 Dengzhuang South Road, Haidian District, Beijing 100094, China

**Keywords:** burst super-resolution, CNN-Transformer, multi-frame super-resolution

## Abstract

Multi-frame super-resolution (MFSR) leverages complementary information between image sequences of the same scene to increase the resolution of the reconstructed image. As a branch of MFSR, burst super-resolution aims to restore image details by leveraging the complementary information between noisy sequences. In this paper, we propose an efficient burst-enhanced super-resolution network (BESR). Specifically, we introduce Geformer, a gate-enhanced transformer, and construct an enhanced CNN-Transformer block (ECTB) by combining convolutions to enhance local perception. ECTB efficiently aggregates intra-frame context and inter-frame correlation information, yielding an enhanced feature representation. Additionally, we leverage reference features to facilitate inter-frame communication, enhancing spatiotemporal coherence among multiple frames. To address the critical processes of inter-frame alignment and feature fusion, we propose optimized pyramid alignment (OPA) and hybrid feature fusion (HFF) modules to capture and utilize complementary information between multiple frames to recover more high-frequency details. Extensive experiments demonstrate that, compared to state-of-the-art methods, BESR achieves higher efficiency and competitively superior reconstruction results. On the synthetic dataset and real-world dataset of BurstSR, our BESR achieves PSNR values of 42.79 dB and 48.86 dB, respectively, outperforming other MFSR models significantly.

## 1. Introduction

As a crucial low-level computer vision task, image super-resolution helps to drive research in advanced tasks such as target detection [1,2] and image segmentation [3], in in addition to reconstructing image details. Traditional image super-resolution methods are mainly based on interpolation and filtering techniques. Although these methods can increase the resolution of the image, they cannot restore the lost detail information. In recent years, with the rise of deep learning, image super-resolution methods based on neural networks have made significant breakthroughs in improving reconstruction accuracy and visual effects [4]. Super-resolution technology has been widely used in video surveillance [5], remote sensing [6], medical image diagnosis [7], and other fields [8,9,10]. In particular, with the increasing popularity and professionalization of mobile photography, burst super-resolution technology is attracting more and more attention. Burst is a common photography mode, where multiple images are captured in rapid succession within a short period of time. Due to camera shake, there exists sub-pixel displacement among these multiple images. Burst super-resolution [11] aims to restore image details by utilizing the complementary information from multiple displaced images.

SRCNN [12] is a three-layer convolutional network, as well as the first attempt at deep learning methods for super-resolution problems. The residual network structure addresses the issue of gradient vanishing that arises from increased network depth, leading to improved convergence and enhanced model performance. VDSR [13] uses residual learning to increase the number of layers of the super-resolution network to 20 for the first time. A generative adversarial network (GAN) consists of a generator and a discriminator. SRGAN [14] introduces GAN into the super-resolution task, enabling the generation of more realistic textures in the reconstruction. ESRGAN [15] and SFTGAN [16] optimize the adversarial loss function and incorporate prior information, respectively. Other convolutional network-based super-resolution algorithms, such as [17,18,19], achieve better training results through deeper and more intricate network structures.

In recent years, Transformer has attracted widespread attention in the field of NLP. Thanks to its parallel computing and long-distance dependency modeling capabilities, it has significantly improved the model’s ability to learn rich semantic representations. ViT [20] introduces a Transformer into the computer vision field and has achieved remarkable success. For pixel-level visual tasks, such as image segmentation and super-resolution, the introduction of the Transformer model enhances the network’s understanding of global semantics and can capture the correlation between different pixels or semantics. TTSR [21] introduces the Transformer model to the super-resolution task for the first time, learning the deep correspondence between the low-resolution (LR) image and the reference image through self-attention, and transferring similar textures in the reference image to restoring the high-resolution (HR) image. However, the original Transformer has a complexity of O(n^2^), which comes with high computational costs.

Currently, in the task of super-resolution, Transformer models with linear complexity mainly fall into two categories. One approach involves computing self-attention within a window, as seen in SwinIR [22], which divides the input image into non-overlapping windows and achieves cross-window information interaction through shifting. HAT [23] introduces overlapped cross-attention modules to further enhance the aggregation of information across windows. Another approach involves implicitly encoding global information across feature dimensions, as demonstrated by Restormer [24], which computes cross-channel covariance to implicitly model global information. SAFMN [25] introduces a convolutional channel mixer to simultaneously encode local context and blend channels, achieving global information interaction. We further optimize the implicit global information encoding method and propose a more efficient self-attention mechanism.

Multiple frames of images are sampled from different sub-pixel positions, exhibiting sub-pixel displacement relationships. Multi-frame super-resolution can additionally leverage inter-frame complementary information, recovering more details compared to single-frame super-resolution, and has broad application prospects in fields such as computational photography [26,27], remote sensing satellite imaging [28,29], etc. Commonly used MFSR datasets include PROBA-V [30] for remote sensing tasks and BurstSR [31] for computational photography tasks. The performance improvement of multi-frame super-resolution relies on the sub-pixel-level alignment of multiple frames, and the accuracy of alignment directly impacts the reconstruction results.

Inter-frame alignment and feature fusion are key challenges in burst super-resolution. Frame alignment methods are mainly divided into two types: one involves explicit frame alignment through optical flow estimation and motion compensation [32], and the other employs deformable convolution to learn dynamic sampling positions of adjacent frames for implicit frame alignment [33]. However, existing methods have certain limitations in extracting spatiotemporal correlation information. Therefore, we propose two optimized modules to extract more complementary information from aligned multi-frame images to recover high-frequency details.

To more effectively capture global information, we propose a gate-enhanced Transformer, Geformer. By computing self-attention along the channel dimension, Geformer can implicitly encode global information while maintaining linear complexity. The gating mechanism enables Geformer to effectively model correlations between domain-specific features. We leverage reference features to facilitate inter-frame communication, simultaneously serving as an implicit image co-registration condition to enhance spatiotemporal information consistency. By combining Geformer with convolution, we construct an enhanced CNN-Transformer block (ECTB), providing enhanced feature representation for the two crucial processes of inter-frame alignment and feature fusion. Consequently, we introduce an efficient burst-enhanced super-resolution network, BESR.

We conduct extensive experiments on synthetic and real-world datasets of BurstSR. The results demonstrate a significant improvement in perceptual quality and detail fidelity compared to previous MFSR methods. In terms of PSNR and SSIM metrics, our BESR achieves a gain of 0.35 dB and 0.06 dB, respectively, over the state-of-the-art method RBSR on the two datasets, while having lower parameters. The extensive experimental results provide strong evidence for the effectiveness of our approach.

The main contributions of this paper are summarized as follows:

1. We propose Geformer, a gate-enhanced Transformer model, and combine Geformer with convolution to construct an enhanced CNN-Transformer block (ECTB), enabling the network to simultaneously perceive global information and local details, thereby achieving enhanced feature representation.
2. We enhance the interaction between inter-frame information and boost the correlation of spatiotemporal features by constructing reference features, providing more effective information for the entire super-resolution process.
3. Addressing the critical issues of inter-frame alignment and feature fusion, we introduce optimized pyramid alignment (OPA) and hybrid feature fusion (HFF) modules to fully leverage non-redundant information between frames. Our BESR achieves the optimal reconstruction performance on both synthetic and real-world datasets of BurstSR.

## 2. Related Work

### 2.1. Single-Image Super-Resolution (SISR)

SRCNN is the first method to use deep learning to construct a nonlinear mapping between LR and HR, which achieves SOTA performance on multiple public datasets with only three convolutional layers. Subsequent research has shown that by increasing network depth and introducing residual learning, such as [13,34] and other methods, image quality and perceptual quality can be further improved. Research on generative adversarial networks (GANs), such as [14,35], is introduced to generate more realistic and detailed high-resolution images through adversarial training. GANs not only enhance quantitative evaluation metrics but also improve subjective perceptual quality.

Recent studies suggest that attention mechanisms, through feature selection, can strengthen the extraction of important information while suppressing less relevant details, thereby enhancing the network’s feature extraction capabilities. For instance, RCAN [36] combines residual learning with channel attention mechanisms and trains a super-resolution network with over 400 layers. HAN [37] introduces layer attention and channel-wise spatial attention modules to learn the direct correlation between layer, channel, and spatial features, resulting in an enhanced feature representation.

Due to the inherent advantage of Transformer in modeling long-distance dependencies, Transformer-based super-resolution models have achieved significant performance improvements. SwinIR [22] divides the input image into windows, computing self-attention between patches within each window and using a moving window to model distant dependencies, thus reducing computational complexity linearly. HAT [23] introduces overlapping cross-attention modules to enhance information interaction between adjacent windows. However, the local windows limit the utilization of crucial global information in image super-resolution. Restormer [24] implicitly encodes global information by computing self-attention across feature channels, resulting in a more lightweight super-resolution network. We have improved its limitations in spatial modeling by introducing a gating mechanism to dynamically modulate self-attention, enabling better modeling of the correlation of neighborhood features and extracting more effective global information.

### 2.2. Multi-Frame Super-Resolution (MFSR)

The research on multi-frame super-resolution is first proposed by Tsai and Huang [38]. Compared with SISR methods, MFSR is able to utilize additional inter-frame complementary information to achieve better high-frequency detail recovery. Farsiu et al. [39] propose a bilateral total variation regularization method based on the total variation super-resolution approach. Wronski et al. [27] utilized kernel regression to align input frames, introduced radial kernels for local adaptive detail enhancement, and achieved mosaic removal through multi-frame super-resolution. Deudon et al. [40] focus on MFSR in satellite imagery, addressing subtasks such as joint learning of registration, fusion, and upsampling. They introduced the first deep learning-based multi-frame super-resolution network, HighRes-net.

Bhat et al. [11] introduce the baseline DBSR for burst super-resolution, aligning multiple frames through optical flow and utilizing attention modules to fuse aligned frame information, addressing real-world MFSR challenges. Later, Bhat et al. [41] optimized the multi-frame fusion mechanism by modeling the image formation process in the latent space and conducting a deep reparameterization of the classical MAP formulation. Dudhane et al. [42] propose a pseudo-burst fusion strategy for inter-frame information interaction, enhancing it with multiscale context. Luo et al. [43] employ optical flow-guided deformable convolution for inter-frame alignment, using Swin Transformer as the backbone network for deep feature extraction and image reconstruction.

These models often come with complex parameters and computational requirements. In comparison, the lightweight MFSR model is more practical. Wu et al. [44] proposed a recursive model, RBSR, which merges inter-frame complementary information frame by frame and introduces implicit weighted loss to handle sequences of varying lengths. We have further improved frame alignment by constructing a feature pyramid to integrate multi-scale aligned features, achieving more precise alignment effects, and providing more complementary information for feature fusion.

### 2.3. Efficient Vision Transformers

In recent years, Vision Transformers have experienced rapid development, demonstrating outstanding performance in various tasks. However, constrained by high computational costs, they are not ideal for real-time applications. Therefore, researchers have started to explore lightweight Vision Transformer models. For example, more efficient attention mechanisms have been introduced into the model, such as sparse attention [45,46,47] and local attention [48,49,50]. CvT [51] uses convolutional mapping instead of position-wise linear projection and undersamples *K* and *V* matrices to improve attention efficiency. Researchers have also explored methods such as model pruning [52] and parameter quantization [53] to compress model size, reducing memory requirements while maintaining high performance. The research on efficient Vision Transformers is expected to provide feasible solutions for scenarios with limited computational resources. In this study, we propose an efficient Transformer model, Geformer. Introducing a gating mechanism enhances the modeling of spatial relationships in the neighborhood, allowing for the implicit encoding of global information with higher computational efficiency.

## 3. Methodology

### 3.1. Network Architecture

**Figure 1:**
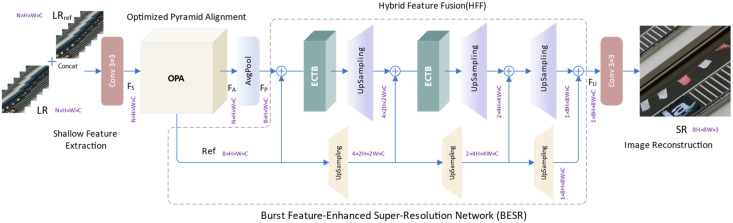
The overall architecture of BESR.

The proposed BESR framework, as illustrated in Figure 1, is primarily based on four key components: shallow feature extraction, optimized pyramid alignment (OPA), hybrid feature fusion (HFF), and image reconstruction.

Specifically, the input of the network is the RAW burst sequences LR, whose size is *N* × *H* × *W* × *C*_0_. Among them, *N*, *H*, *W*, and *C*_0_, respectively, represent the frames, height, width, and channels of the LR. The specific values are 14, 48, 48, and 4, respectively. The output of the super-resolution network is a high-resolution RGB image SR with a size of 8*H* × 8*W* × 3. We generate the reference feature by repeating the 0-th frame of the input feature *N* times, and then concatenate the input feature and the reference feature along the channel dimension to obtain the feature $F_{C}$, whose size is *N* × *H* × *W* × 2*C*_0_.

(1) $${\mathrm{LR}}_{\mathrm{ref}}\left[i]=\mathrm{LR}\;[0],\;i\in [0,N-1\right]$$

(2) $$F_{C}[n,h,w,c]=\left\{\begin{array}{c} \mathrm{LR}[n,h,w,c],\;c\in [0,C-1]\\ {\mathrm{LR}}_{\mathrm{ref}}\left[n,h,w,c-C\right],\;c\in [C,2C-1] \end{array}\right.$$

where *i* denotes the *i*-th frame of the reference feature. We employ a single layer of 3 × 3 convolution to aggregate information from concatenated features, mapping them to a high-dimensional feature representation, thus obtaining shallow feature $F_{S}$ with a size of *N* × *H* × *W* × *C*. Here, *C* represents the channels in the intermediate features, which we set to 48.

(3) $$F_{S}{=Conv}_{3}{(F}_{C})$$

where ${Conv}_{3}$ represents a 3 × 3 convolution. The enhancement of reference features can not only promote information interaction between frames, but also serve as an implicit multi-frame co-registration condition, helping to reduce the impact of alignment errors.

In the optimized pyramid alignment (OPA) module, we employ a combination of deformable convolution and enhanced CNN-Transformer blocks (ECTB) for multi-scale feature alignment. Subsequently, through the refinement of a cascaded alignment network, we can capture fine-grained inter-frame displacements, achieving more precise feature alignment and obtaining enhanced aligned features, denoted as $F_{A}$, with dimensions *N* × *H* × *W* × *C*.

(4) $$F_{A}{=\mathrm{OPA}(F}_{S})$$

Our BESR can flexibly handle different numbers of inputs. To capture more effective information from the inputs, we use all 14 bursts from the same scene as the input to the model. For convenience in subsequent feature transformations, we adjust the size of the aligned features by applying the average pooling operator in the AvgPool module to obtain the pooled features $F_{P}$ as the input to the fusion module, with a size of 8 × *H* × *W* × *C*.

(5) $$F_{P}{=\mathrm{AvgPool}(F}_{A})$$

In the hybrid feature fusion (HFF) module, we integrate inter-frame complementary information using ECTBs and facilitate inter-frame communication with reference features, enhancing the spatiotemporal correlations among multiple frames. Subsequently, we combine 1 × 1 convolution and Pixel Shuffle to upsample the fused features, achieving the restoration of high-frequency details. Finally, through a 3 × 3 convolution layer, we map the fused high-level semantic feature $F_{U}$ (with dimensions 8*H* × 8*W* × C) to a high-resolution RGB image SR, sized 8*H* × 8*W* × 3.

(6) $$F_{U}{=\mathrm{HFF}(F}_{P})$$

(7) $${\mathrm{SR}=Conv}_{3}{(F}_{U})$$

### 3.2. Enhanced CNN-Transformer Block

**Figure 2:**
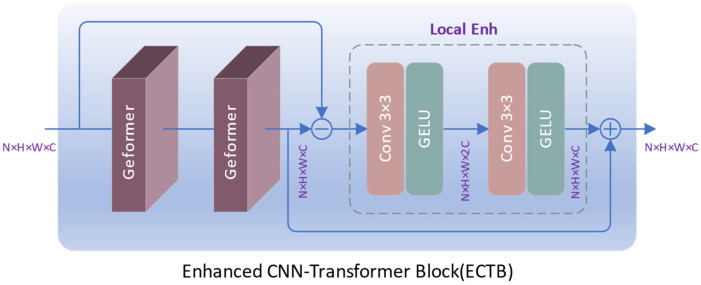
The structure of ECTB.

For pixel-level visual tasks such as image segmentation and super-resolution, when the input image size is *H* × *W* pixels, the complexity of the Transformer model is O(*H*^2^*W*^2^). The quadratic complexity results in high computational costs. In super-resolution tasks, the key lies in capturing similar features. Features in spatial proximity exhibit higher similarity, while those at spatially distant locations demonstrate lower similarity. Standard Transformers acquire attention maps through global computations, which results in a large number of redundant calculations. At the same time, it also lacks local perception capabilities and does not capture high-frequency information well. To solve these problems, we propose an enhanced CNN-Transformer block (ECTB), as shown in Figure 2.

(8) $$X_{F}=\mathrm{Geformer}(\mathrm{Geformer}(X))$$

(9) $$X_{E}{=X}_{F}{+\mathrm{Local}\;\mathrm{Enh}(X}_{F}\;-\;X))$$

Here, X represents the input feature, while $X_{F}$ and $X_{E}$, respectively, denote the intermediate and output features of ECTB, all with dimensions *H* × *W* × *C*. By adopting a CNN-Transformer hybrid structure, ECTB can simultaneously perceive global context and local detailed features, resulting in an enhanced feature representation.

#### 3.2.1. Geformer

**Figure 3:**
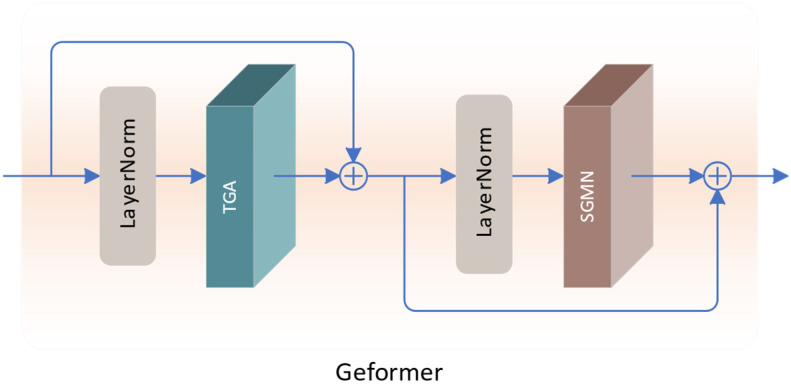
The structure of Geformer.

Inspired by Restormer [24], we model global correlations by computing the covariance across feature channels, and integrate gating mechanisms in the attention and FFN components to model neighborhood correlations. Consequently, we propose a gate-enhanced Transformer called Geformer, whose structure is illustrated in Figure 3. It comprises two core components: Transposed Gating Attention (TGA) and Spatial-Gate Modulated Network (SGMN).

(10) $$X_{N}{=X}_{in}{+\mathrm{TGA}(LN(X}_{in}))$$

(11) $$X_{M}{=X}_{N}{+\mathrm{SGMN}(LN(X}_{N}))$$

Among them, $X_{in}$ represents the input feature, while $X_{N}$ and $X_{M}$ represent the intermediate and output features of Geformer, respectively, and their sizes are *H* × *W* × *C*. *LN* represents the LayerNorm layer. Through the introduced gating mechanism, Geformer achieves dynamic spatial modulation of self-attention, which can better capture the correlation of neighborhood features and provide more effective contextual information for super-resolution tasks.

**Figure 4:**
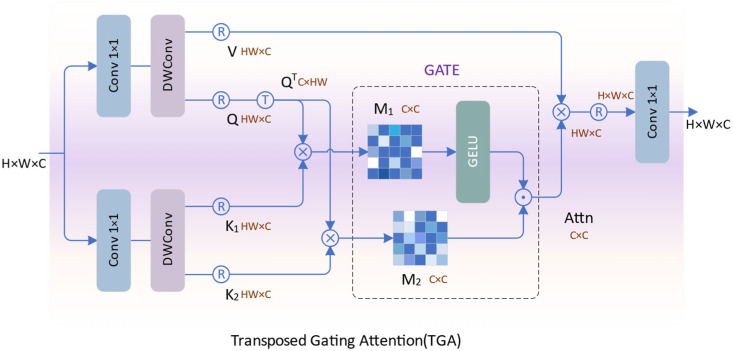
The illustration of TGA.

Transposed Gating Attention (TGA) is a gate-enhanced self-attention mechanism we proposed, as illustrated in Figure 4. It enhances the ability to extract global correlations by calculating cross-covariance across feature channels and introducing a gating mechanism to model spatial neighborhood correlations.

Specifically, given an input feature $X_{0}$ of size *H* × *W* × *C*, we expand its channels through two 1 × 1 convolutions. Subsequently, we enhance local contextual awareness through a 3 × 3 depth-wise separable convolution. After reshaping the features, we obtain the query projection (*Q*), the value projection (*V*), and two key projections ($K_{1}$ and $K_{2}$), where *Q*, *V*, $K_{1}$, and $K_{2}$ are all of size *HW* × *C*.

(12) $${Q,V=Split(DWConv}_{31}{(Conv}_{11}{(X}_{0})))$$

(13) $$K_{1}{,K}_{2}{=Split(DWConv}_{32}{(Conv}_{12}{(X}_{0})))$$

Among them, ${Conv}_{11}$ and ${DWConv}_{31}$ represent the first 1 × 1 convolution and 3 × 3 depth-separable convolution, and ${Conv}_{12}$ and ${DWConv}_{32}$ represent the second 1 × 1 convolution and 3 × 3 depth-separable convolution. By transposing *Q*, we obtain the transposed projection $Q^{T}$ with dimensions *C* × *HW*. We perform element-wise multiplication between $Q^{T}$ and $K_{1}$ as well as $Q^{T}$ and $K_{2}$ to assess the similarity between the input features, yielding two weight matrices, $W_{1}$ and ${\;W}_{2}$. Subsequently, we apply GELU to activate $W_{1}$, serving as a gating modulation unit for $W_{2}$. By element-wise multiplication of two matrices and applying softmax mapping, the modulated attention map *Attn* is obtained. Then, it is multiplied with *V* to enhance similar features. Finally, through 1 × 1 convolution, the TGA produces output features $X_{A}$ with a size of *H* × *W* × *C*.

(14) $$W_{1}=Q^{T}⊗K_{1}{,\;W}_{2}=Q^{T}⊗K_{2}$$

(15) $${Attn=Softmax(\mathrm{GELU}(W}_{1}{)\cdot W}_{2}/\alpha )$$

(16) $$X_{A}{=Conv}_{1}(V⊗Attn)$$

Here, *α* is a learnable scaling parameter, and ⊗ represents matrix multiplication. Compared to the original self-attention, TGA can effectively capture the correlations between spatial neighborhood features, enhancing the exploration of global information.

**Figure 5:**
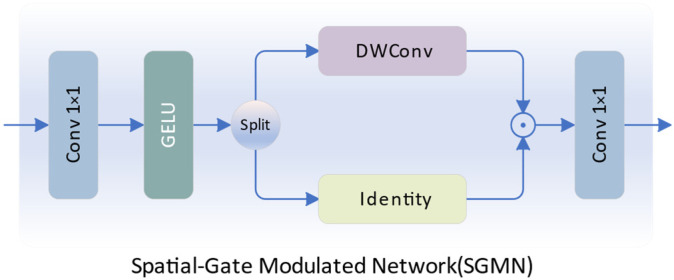
The illustration of SGMN.

The Feedforward Neural Network (FFN) introduces non-linearity to the Transformer, but its limitations in feature fitting arise due to the implicit modeling of spatial and channel relationships. Therefore, we propose the Spatial-Gate Modulated Network (SGMN), a gate-enhanced efficient feedforward network, as illustrated in Figure 5.

Specifically, the network takes the enhanced feature $X_{T}$ obtained from TGA as input. We perform high-dimensional mapping on $X_{T}$ using a 1 × 1 convolution, followed by nonlinear activation with GELU. Along the channel dimension, we partition the features into two parts, resulting in two features $X_{1}$ and $X_{2}$ of size *H* × *W* × *C_h_*, where *C_h_* denotes the channels in the high-dimensional feature mapping. One branch undergoes local contextual enhancement via depth-wise separable convolution, while the other branch undergoes an identity mapping. Spatial modulation is achieved by element-wise multiplication of these two features. Finally, we aggregate information along the spatial dimension using a 1 × 1 convolution to obtain the nonlinearly enhanced feature representation $X_{NL}$ with a size of *H* × *W* × *C*.

(17) $$X_{1}{,X}_{2}=Split(\mathrm{GELU}({Conv}_{1}{(X}_{T})))$$

(18) $$X_{NL}{=Conv}_{1}{(DWConv}_{3}{(X}_{1}{)\cdot X}_{2})$$

Compared with standard FFN, our SGMN can explicitly model spatial information while reducing redundant information between channels and has a smaller number of parameters.

Compared to existing Transformer models, our proposed Geformer maintains linear instead of quadratic computational complexity by establishing inter-feature channel information interaction. Benefiting from the gating mechanisms introduced in TGA and SGMN, Geformer can model spatial neighborhood correlations while capturing global information, thus providing powerful feature learning capabilities for super-resolution reconstruction tasks.

#### 3.2.2. Local Enhancement

To enhance the perception of local features, we construct a local enhancement (Local Enh) module (as shown in Figure 2) for extracting local residuals, which are then added to the output of the Geformer to obtain augmented hybrid features. The Local Enh module comprises two cascaded layers of 3 × 3 convolutions and GELU, designed to excite and compress features, thereby capturing more local detail information. Given an input feature $X_{G}$, the output feature $X_{C}$ of Local Enh can be expressed as:

(19) $$X_{C}{=\mathrm{GELU}(Conv}_{3}{(\mathrm{GELU}(Conv}_{3}{(X}_{G}))))$$

The first layer of convolution doubles the channels in the feature map, while the second layer of convolution recovers the number of channels by reduction. The size of both $X_{G}$ and $X_{C}$ is *H* × *W* × *C*.

### 3.3. Optimized Pyramid Alignment

Compared with SISR, MFSR can utilize the complementary information between frames by aligning multiple frames with sub-pixel displacements to provide more effective information for the reconstruction of high-frequency details of the image. Inter-frame alignment is a key issue in multi-frame super-resolution. Effective alignment can strengthen the spatiotemporal correlation between frames, allowing the model to capture sub-pixel differences between frames. Enhancing through the fusion of adjacent frame information is helpful in mitigating motion-induced blurriness and distortion.

Deformable alignment uses deformable convolution to estimate the offset and resample adjacent frames to achieve alignment with the reference frame. Deformable convolution extends traditional convolution by introducing a learnable offset for each convolution sampling point to adjust the sampling points at each position in the convolutional kernel. These offsets are generated by the input feature map and another convolution.

Specifically, given a convolutional kernel with *K* sampling positions, where $w_{k}$ and $n_{k}$ represent the weight and pre-set offset of the *k*-th position, and let *x*(*n*) and *y*(*n*) denote the features at position *n* in the input feature map *x* and output feature map *y*, deformable convolution can be defined as follows:

(20) $$\;y=\overset{K}{\underset{k=1}{\sum }}w_{k}\cdot x\left({n+n}_{k}{+\Delta n}_{k}\right)\cdot {\Delta m}_{k}$$

where ${\Delta n}_{k}$ and ${\Delta m}_{k}$ represent the learnable offset and modulation scalar at the *k*-th position, with ${\Delta m}_{k}$ constrained within the range [0, 1]. As the coordinates after incorporating ${\Delta n}_{k}$ are typically non-integer, resulting in irregular sampling positions, we employ bilinear interpolation to resample the feature map and obtain the displaced features.

**Figure 6:**
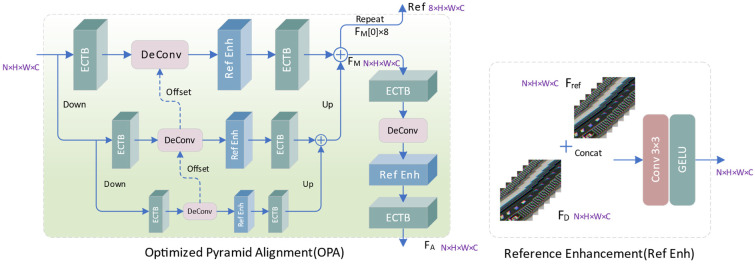
The structure of OPA and Ref Enh.

Based on deformable convolution, we propose an optimized pyramid alignment (OPA) module, as shown in Figure 6. In each layer of the alignment network, we first aggregate intra-frame context and inter-frame correlation information through the proposed ECTB to obtain enhanced feature representation, and then utilize deformable convolution to perform motion estimation and compensation on the aggregated features. In the reference enhancement (Ref Enh) module, we concatenate and fuse the input feature $F_{D}$ and the reference feature $F_{ref}$ along the channel dimension to promote inter-frame communication and enhance the mining of spatiotemporal associated information.

(21) $$F_{C}^{\prime }[n,h,w,c]=\left\{\begin{array}{c} F_{D}\left[n,h,w,c],\;c\in [0,C-1\right]\\ F_{ref}\left[n,h,w,c-C\right],\;c\in [C,2C-1] \end{array}\right.$$

(22) $$F_{R}{=\mathrm{GELU}(Conv}_{3}{(F}_{C}^{\prime }))$$

where $F_{C}^{\prime }$ and $F_{R}$ represent concatenated features and fused features, respectively. OPA is a top-down three-level feature pyramid, where each alignment network layer consists of ECTBs on both sides, along with a deformable convolution and a reference enhancement module. To generate the input feature at the (*i* + 1)-th level, we utilize a convolution with a 3 × 3 kernel and a stride of 2 to downsample the input at the *i*-th pyramid level.

(23) $$F_{i+1}{=Conv}_{S2}{(F}_{i})$$

where $F_{i}$ and $F_{i+1}$ represent the input features of *i*-th level and (*i* + 1)-th level, respectively. The input of the *i*-th deformable convolution consists of two parts: the refined feature output by the *i*-th level ECTB and the offset output by the (*i* + 1)-th level deformable convolution. The aligned features and offsets output by the (*i* + 1)-th level are upsampled through Pixel Shuffle and deconvolution, respectively, and then input to the *i*-th level. For each level of alignment network,

(24) $$F_{i}^{\prime }{=\mathrm{ECTB}}_{R}{(\mathrm{Ref}\;\mathrm{Enh}(DeConv(ECTB}_{L}{(F}_{i}))))$$

(25) $$F_{M}{=\mathrm{Up}(\mathrm{Up}(F}_{2}^{\prime }{)+F}_{1}^{\prime }{)+F}_{0}^{\prime }$$

Among them, $F_{i}^{\prime }$ represents the output features and alignment features of the *i*-th level. And ${\mathrm{ECTB}}_{L}$ and ${\mathrm{ECTB}}_{R}$, respectively, represent the ECTB blocks on both sides of each layer of the aligned network. By integrating the output of the three-layer network, we obtain the multi-scale aligned feature $F_{M}$. By repeating the 0-th frame of $F_{M}$ 8 times, we construct reference features to provide more contextual information for feature fusion.

(26) $$Ref[i]=F_{M}[0],\;i\in [0,7]$$

To further enhance the spatiotemporal consistency of inter-frame features, we cascade another layer of alignment network after the pyramid structure to refine the $F_{M}$ and obtain an enhanced alignment feature $F_{A}$, which further improves the alignment accuracy and can provide more supplementary information for reconstruction.

(27) $$F_{A}{=\mathrm{ECTB}}_{R}{(\mathrm{Ref}\;\mathrm{Enh}(DeConv(ECTB}_{L}{(F}_{M}))))$$

### 3.4. Hybrid Feature Fusion

**Figure 7:**
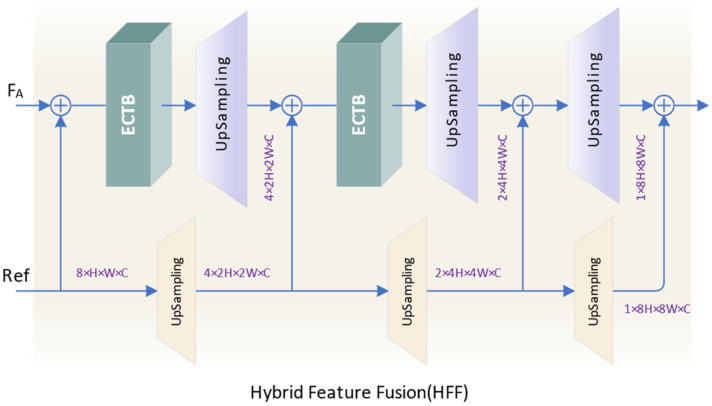
The structure of HFF.

For feature fusion, we propose a hybrid feature fusion module (HFF), as shown in Figure 7. The three-layer network enables the model to extract more effective information from the fused features. We use reference features to enhance inter-frame correlations and add them to the aligned features as input to the fusion module. The introduction of reference features provides additional reference information for feature fusion while facilitating the interaction of interframe information. By introducing an ECTB to capture and aggregate global and local contextual information in input features, the network can more fully exploit inter-frame complementary information, resulting in finer high-frequency detail recovery.

In HFF model, the input of the (*i* + 1)-th level network is obtained by adding the output features of the *i*-th level network and the reference features. At each level of the network, the input features are fused with complementary information between frames through ECTB. We then utilize 1 × 1 convolution for channel expansion and upsampling via pixel shuffling to recover the lost details.

(28) $$Y_{i+1}{=\mathrm{Pixel}\;\mathrm{Shuffle}(Conv}_{1}{(\mathrm{ECTB}(Y}_{i}{+Ref}_{i})))$$

where $Y_{i}$ and ${Ref}_{i}$ represent the output feature and reference feature of the *i*-th level, respectively. And $Y_{i+1}$ represents the output feature of the (*i* + 1)th-level. The stepwise feature fusion and upsampling strategy helps to preserve the spatial information of the image while mitigating the impact of noise and blurring factors, thereby enhancing the reconstruction effect. To reduce the model’s parameters, we use ECTB only in the first two layers and share parameters between the three upsampling layers of reference features to achieve a lightweight model while maintaining high performance.

## 4. Experiments

### 4.1. BurstSR Dataset

BurstSR is a benchmark for the MFSR task proposed by the NTIRE 2022 Burst Super-Resolution Challenge, which includes synthetic and real-world datasets. Among them, the synthetic dataset consists of 46,839 bursts for training and 300 bursts for validation. Each burst contains 14 RAW images of size 48 × 48 pixels. These images are obtained by converting the raw images from sRGB to linear space by inverse camera pipeline, then randomly translating, rotating, and downsampling each image by bilinear interpolation, and finally, mosaicking using Bayer mode. The real-world dataset includes 5405 bursts for training and 882 bursts for validation. The LR images and HR images are captured by a smartphone and a DSLR camera with a zoom lens, respectively. They exhibit certain misalignments in both spatial and color aspects. Therefore, we employ aligned L1 loss and perceptual loss for model training and evaluate the model performance using aligned PSNR and SSIM.

### 4.2. Evaluation Metrics

Quantitative evaluation based on objective assessment methods is the mainstream evaluation metric in the current field of super-resolution reconstruction. Among them, peak signal-to-noise ratio (PSNR) and structural similarity (SSIM) are two of the most commonly used metrics for image quality evaluation. PSNR is the most widely used image reconstruction quality evaluation metric in the current task of super-resolution reconstruction. It measures the quality of an image by calculating the differences between corresponding pixels in the reconstructed image and the ground truth image. A higher PSNR value indicates less distortion in the reconstructed image. SSIM is a full-reference image quality assessment metric used to measure the similarity between a reconstructed image and a ground truth image in terms of brightness, contrast, and structure. It provides a more accurate reflection of human perception of image quality, and a value closer to 1 indicates higher similarity between the reconstructed image and the ground truth image. Learned Perceptual Image Patch Similarity (LPIPS) is a deep learning-based image quality assessment metric. It evaluates the quality of images by learning human perception, where a lower value indicates greater similarity between two images. We use LPIPS along with PSNR and SSIM as evaluation metrics for the super-resolution models.

### 4.3. Training Details

We choose the Adam optimizer for model training and set the decay rate parameters *β*_1_ and *β*_2_ to 0.9 and 0.999. During model training, we use the L1 norm as the loss function and set the batch size to 4. The learning rate is gradually reduced during the training phase according to the decay strategy of cosine annealing, from the initial value of 1 × 10^−4^ to 1 × 10^−6^. We trained the proposed BESR model for 300 epochs using 4 NVIDIA A5000 GPUs (NVIDIA, Beijing, China) based on the PyTorch 1.10.2 framework. For real-world data, we use the model weights trained on synthetic data to fine-tune it, with a period of 75 epochs, and the learning rate is reduced from 1 × 10^−5^ to 3 × 10^−6^.

### 4.4. Comparison with State-of-the-Art Methods

#### 4.4.1. Quantitative Results

We quantitatively compare the proposed BESR with other state-of-the-art MFSR methods, including DBSR, HighRes-net, MFIR, BIPNet, BSRT-S, and RBSR, on benchmark synthetic datasets and real-world datasets. We use PSNR and SSIM as objective evaluation indicators of the reconstruction performance of different methods and report the parameters and inference time of the models, and the comparison results are shown in Table 1. The time in the table represents the inference time required to generate a single SR image of size 384 × 384.

The comparison results indicate that, compared to other MFSR methods, our BESR achieves the best reconstruction results on almost all evaluation metrics, fully demonstrating the effectiveness of the proposed approach. Thanks to the efficient self-attention mechanism in Geformer, BESR maintains lower model parameters compared to other methods while enhancing performance. By adopting a CNN-Transformer hybrid structure, our model can simultaneously capture local details and global context, providing more effective information for the super-resolution task. Compared to the latest model RBSR, our method achieves a PSNR gain of 0.35 dB and 0.06 dB on the synthetic dataset and real-world dataset of BurstSR, respectively, while also obtaining competitive LPIPS results.

#### 4.4.2. Visual Comparisons

**Figure 8:**
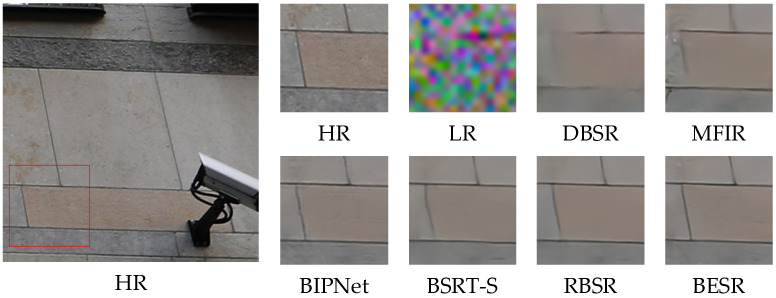
Visual comparison of img_0012 on the synthetic dataset.

**Figure 9:**
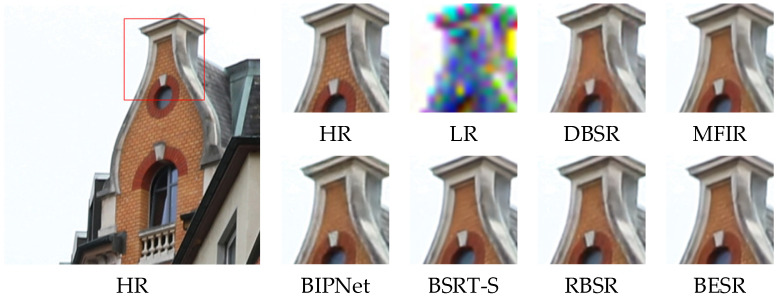
Visual comparison of img_0060 on the synthetic dataset.

**Figure 10:**
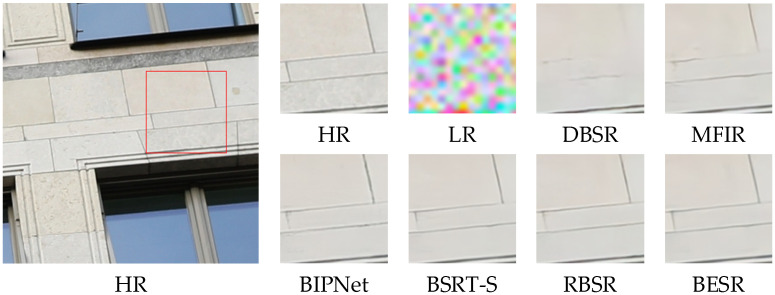
Visual comparison of img_0116 on the synthetic dataset.

**Figure 11:**
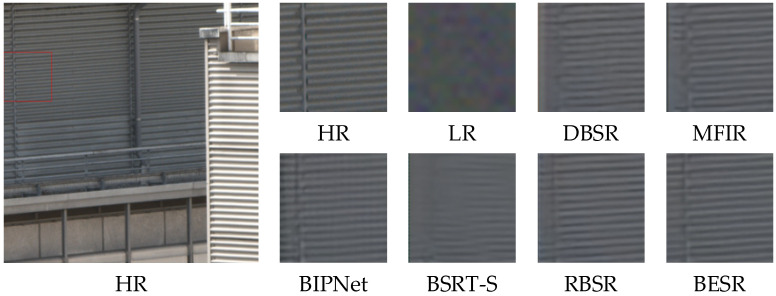
Visual comparison of img_0010_0013 on the real-world dataset.

**Figure 12:**
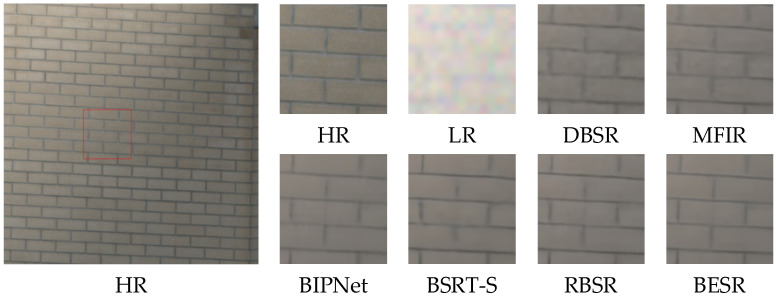
Visual comparison of img_0060_0015 on the real-world dataset.

**Figure 13:**
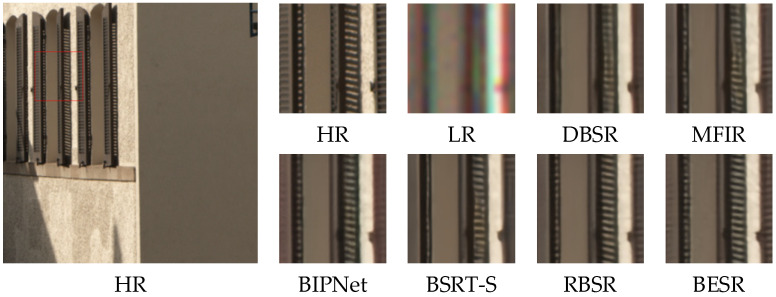
Visual comparison of img_0077_0012 on the real-world dataset.

The visual results of the synthetic and real-world datasets are shown in Figure 8, Figure 9 and Figure 10 and Figure 11, Figure 12 and Figure 13, respectively. The red box represents the target area that we have selected. Comparison results across different scenes indicate that, compared to other MFSR methods, our proposed BESR can better restore high-frequency details lost in the images and exhibit better robustness to noise, resulting in visually appealing HR images.

For example, as shown in Figure 8 and Figure 10, our super-resolution results reconstruct sharp and natural edges, while the generated results from other methods exhibit significant texture distortions. As depicted in Figure 9, our method can recover more details of the wall, capturing relatively complete local structures, whereas the reconstruction results from other methods are blurry and suffer from severe detail distortions. These results demonstrate that our method effectively restores more high-frequency information.

From the visual comparisons in Figure 11 and Figure 13, it is evident that the reconstruction results from other methods exhibit significant blurring and detail distortions, whereas our reconstruction results have fewer artifacts and sharper texture details. Especially in the scene img_0077_0012, only our method effectively restores the details of the windows. In Figure 12, it can be observed that our reconstructed results have sharper lines and higher clarity.

The visual comparison results indicate that our method, while enhancing the overall structure, also focuses on local texture details, resulting in better super-resolution results, further demonstrating the exceptional reconstruction performance of the proposed method.

#### 4.4.3. Ablation Study

In this section, we discuss the effectiveness of key modules in BESR, including the enhanced CNN-Transformer block, the optimized pyramid alignment module, and the hybrid feature fusion module. We select the corresponding baselines for the ablation study of the proposed modules and report the results of the ablation experiments on the benchmark dataset in Table 2, Table 3, Table 4 and Table 5. ✓ and ✗ in the table indicate the presence or absence of the module respectively.

- Effectiveness of ECTB.

We use the Transformer Block proposed in Restormer as the corresponding baseline for the core component ECTB in our network. Our proposed Geformer can dynamically modulate self-attention through a gating mechanism, providing better modeling of the correlation of neighborhood features. ECTB adopts a CNN-Transformer hybrid structure, which can effectively capture and process both local and global information in the image, thereby providing more effective information for the recovery of high-frequency details.

Compared to the baseline, ECTB is able to obtain enhanced feature representations, resulting in a 0.47 dB gain in PSNR for improved reconstruction performance. We report the impact of different core components on the model’s reconstruction performance in Table 3. The results show that our model maintains high performance while having lower parameters.

- Effectiveness of OPA.

We chose to use the PCD alignment module proposed in EDVR [55] as the baseline for the OPA module ablation study. By adopting a CNN-Transformer hybrid structure, ECTB has the ability to simultaneously perceive local features and model global information and can effectively capture the spatiotemporal correlation of multi-frame features. In Table 4, ${\mathrm{ECTB}}_{L}$ and ${\mathrm{ECTB}}_{R}$, respectively, represent the ECTBs on the left and right sides of each layer in the alignment network, playing a crucial role in improving the alignment effect. The OPA module performs inter-frame alignment at different scales and obtains more refined alignment features through aggregation. We fuse the reference frame features with the current frame features to further enhance the correlation of inter-frame features, thereby extracting more inter-frame complementary information. Compared with PCD, the OPA module brings a PSNR gain of 1.13 dB to improve model performance.

- Effectiveness of HFF.

We construct a baseline for HFF module ablation research using three upsampling layers consisting of 1 × 1 convolution and Pixel Shuffle. The ECTB proposed in this study can perform fine-grained feature fusion on the obtained alignment features to fully utilize the complementary information between frames for high-frequency detail reconstruction. In addition, the introduction of reference features helps to extract more different information from aligned features, providing an additional reference for reconstruction while alleviating the problem of edge information loss in image features. Compared with the baseline, the model using two layers of ECTB achieved a PSNR gain of 0.68 dB, demonstrating its effectiveness for feature fusion.

## 5. Conclusions

In this study, we propose an efficient burst-enhanced super-resolution network, BESR. Based on the efficient Geformer introduced in the paper, we construct an enhanced CNN-Transformer block that effectively aggregates intra-frame context and inter-frame correlation information from multiple frames. Additionally, we leverage reference features to facilitate inter-frame communication, enhancing spatiotemporal coherence among multiple frames. To address the challenges of inter-frame alignment and feature fusion, we introduce optimized pyramid alignment and hybrid feature fusion modules to extract and utilize complementary information between frames, providing more effective information for the restoration of high-frequency details. Extensive experiments conducted on two benchmark datasets demonstrate that BESR achieves state-of-the-art reconstruction performance, producing high-resolution images with rich details and clear textures. In the future, our work will be dedicated to researching super-resolution algorithms for unsupervised models.

## Figures and Tables

**Table 1:** Quantitative comparison with state-of-the-art MFSR methods on benchmark datasets. Red and blue represent the best and second-best reconstruction performance, respectively.

Methods	Parameters (M)	Time (ms)	Synthetic			Real-World		
			PSNR	SSIM	LPIPS	PSNR	SSIM	LPIPS
SingleImage [11]	13.01	40	36.86	0.919	0.113	46.60	0.979	0.039
HighResNet [40]	34.78	46.3	37.45	0.924	0.106	46.64	0.980	0.038
DBSR [11]	13.01	431	39.17	0.946	0.081	47.70	0.984	0.029
MFIR [41]	12.13	420	41.55	0.964	0.045	48.32	0.985	0.023
BIPNet [42]	6.67	130	41.93	0.967	0.035	48.49	0.985	0.023
BSRT-S [43]	4.92	198	42.72	0.971	0.031	48.48	0.985	0.021
RBSR [44]	6.42	336	42.44	0.970	0.035	48.80	0.987	0.022
BESR (Ours)	3.81	61	42.79	0.971	0.031	48.86	0.987	0.022

**Table 2:** The ablation study of the enhanced CNN-Transformer block (ECTB).

Components	Baseline					
TGA	✗	✗	✓	✓	✓	✓
SGMN	✗	✗	✗	✗	✓	✓
Local Enh	✗	✓	✓	✗	✗	✓
Benchmark	Metrics (PSNR/SSIM)					
Synthetic	42.32/0.968	42.64/0.969	42.71/0.970	42.47/0.968	42.58/0.969	42.79/0.971
Real-world	48.43/0.985	48.67/0.986	48.78/0.986	48.52/0.986	48.64/0.986	48.86/0.987

**Table 3:** Comparison of different core blocks of the network.

Block	Parameters (M)	Synthetic	Real-World
		PSNR/SSIM	PSNR/SSIM
ECTB	42.79/0.971	48.86/0.987	3.81
RSTB [22]	42.75/0.971	48.81/0.986	6.09
RHAG [23]	42.76/0.971	48.83/0.987	7.33
PAB [54]	42.69/0.970	48.77/0.986	5.78

**Table 4:** The ablation study of the optimized pyramid alignment (OPA) module.

Components	Baseline					
${\mathrm{ECTB}}_{L}$	✗	✗	✓	✓	✓	✓
${\mathrm{ECTB}}_{R}$	✗	✗	✗	✗	✓	✓
Ref Enh	✗	✓	✓	✗	✗	✓
Benchmark	Metrics (PSNR/SSIM)					
Synthetic	41.66/0.964	41.78/0.966	42.32/0.968	42.23/0.967	42.64/0.970	42.79/0.971
Real-world	47.93/0.983	48.08/0.984	48.47/0.986	48.35/0.985	48.77/0.987	48.86/0.987

**Table 5:** The ablation study of the hybrid feature fusion (HFF) module.

Components	Baseline					
*N*_ECTB_	0	1	0	1	2	2
Ref	✗	✗	✓	✓	✗	✓
Benchmark	Metrics (PSNR/SSIM)					
Synthetic	42.11/0.966	42.34/0.970	42.18/0.967	42.40/0.969	42.67/0.970	42.79/0.971
Real-world	48.21/0.985	48.43/0.986	48.25/0.985	48.47/0.986	48.79/0.987	48.86/0.987

## Data Availability

The data presented in this study are available on request from the corresponding author.
